# Finding Mr. Right: Housing Quality Affects Male Mouse Attractiveness to Females, With Implications for Conservation Captive Breeding

**DOI:** 10.1002/zoo.70076

**Published:** 2026-05-05

**Authors:** Prathipa Anandarajan, Jiayi Cai, Georgia Mason

**Affiliations:** ^1^ Department of Integrative Biology University of Guelph Guelph Ontario Canada

**Keywords:** conservation captive breeding, female mate choice, housing conditions, male attractiveness, mice

## Abstract

Females generally prefer mates with traits indicating low stress (e.g., large size; good health). In captivity, stress from suboptimal housing might therefore reduce male attractiveness. We tested this hypothesis using mice (*Mus musculus*), predicting that compared to males conventionally housed (CH) in small lab cages, females will preferentially court higher welfare males from well‐resourced (WR) ‘enriched’ conditions. First, a small‐scale pilot opportunistically used 12 DBA/2 males and 22 DBA/2 females, both sexes differentially raised (half CH, half WR), but with heterogeneous reproductive experience. In T‐maze mate choice tests, CH females preferred males from WR housing, spending significantly more time near and sniffing them. Next, the main study used 12 dyads of DBA/2 male litter‐mates, differentially raised from 4 to 12 weeks old. Twelve virgin oestrous DBA/2 female sister pairs were each presented with two differentially raised virgin brothers. Ultrasonic recording confirmed courtship singing in all trials. Choice behaviour was videoed and analyzed blind to housing. Females spent on average 1.5 times longer near males from WR housing than their CH brothers, and sniffed them for nearly twice as long: significant preferences that did not vary with female housing. Effects were not explained by male body weight, testis weight or anogenital distance. Overall, as predicted, WR housing thus rendered males more attractive. When subsequently housed for mating in male‐sister‐pair trios, (6 WR and 6 CH), the WR trios also tended to be more likely to have litters. For species with problematic breeding programmes, improving welfare via higher‐quality housing may therefore help improve reproductive success.

## Summary

- Female mice prefer well‐resourced (‘enriched’) males over conventionally‐housed males.
- Allowed to reproduce, well‐resourced breeding trios also tended to have more pups.
- Effects were not due to male body weight, testis weight, nor anogenital distance.

## Graphical abstract

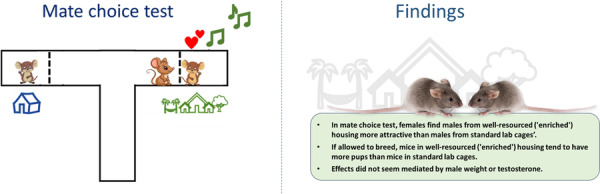

## 1 Introduction

In many species, females have evolved to choose carefully between potential mates, generally being the pickier sex (Trivers 1972; Clutton‐Brock et al. 1981; Kenrick et al. 1990). This is because females must guard their reproductive resources more than males: they invest more into each gamete, and often provide more parental care (Trivers 1972; Clutton‐Brock et al. 1981; Kenrick et al. 1990). In their natural environments, choosing a high‐quality mate thus benefits females' overall reproductive success, doing so both *directly*, by improving their survival or fecundity (via better territory, resources or parental care), and *indirectly* by improving offspring fitness via these beneficial traits (Williams 1975; Taylor and Williams 1982; Reynolds and Gross 1990; Kirkpatrick and Ryan 1991). Consequently, females have evolved to utilize various cues to assess male quality when choosing mates. Females prefer condition‐dependent cues indicative of good nutritional status and low stress (e.g., good health, heavier body weights, brighter coloration, more intricate ornamentation, more complex songs: Parsons 1998; Forstmeier 2002; Adrian et al. 2008; Clutton‐Brock and McAuliffe 2009), and thence better territories or access to resources, or good genotypes that can be passed to offspring (Jennions and Petrie 1997).

The implications of these mate‐choice dynamics extend beyond the wild into captive conditions, because suboptimal housing (e.g., crowded, small and/or barren conditions) can adversely affect the male cues that females prefer. For example, females sometimes prefer cues indicating high testosterone, such as large testes (Jones et al. 2015) and long anogenital distances, AGDs (Ophir and Delbarco‐Trillo 2007), since high testosterone may be associated with the potential for more vigorous or competitive offspring; yet testosterone is stress‐sensitive (e.g., Deviche et al. 2010) and can therefore be reduced in males housed in small, barren cages compared to those in large, well‐resourced (WR) ‘enriched’ ones (e.g., mice: Nevison et al. 1999; American mink [*Neogale vison*]: Díez‐León et al. 2013). Females also often prefer males with relatively low levels of circulating glucocorticoids (e.g., zebra finches [*Taeniopygia guttata*], Roberts et al. 2007; and even humans [Moore et al. 2011]). However, crowded, small and/or barren housing conditions can elevate this hormone (e.g., North American clouded leopard [*Neofelis nebulosa*]: Wielebnowski et al. 2002; red pandas [*Ailurus fulgens*]: Khan et al. 2023). Turning to physical cues, females often prefer symmetrical males, as seen in zebra finches (Swaddle and Cuthill 1994) and again humans (Koehler et al. 2002; Gangestad et al. 2010), because symmetry indicates abilities to cope with challenging conditions (e.g., Swaddle and Cuthill 1994; Özener 2010). In sub‐optimal housing, however (e.g., grid floors and single housing for Lewis rats: Sørensen et al. 2005; small, barren cages for mink: Díez‐León et al. 2013) male symmetry declines. Finally, females often prefer larger or heavier males (e.g., Forstmeier 2002). But again, perhaps because weight can decline with stress (e.g., Jean‐Faucher et al. 1981), males housed in small, barren cages may become smaller and lighter those in large, WR ‘enriched’ ones (e.g., American mink: Díez‐León et al. 2013).

Given such examples, poor male welfare may well reduce their success with females. It is thus not surprising that, in the few studies to investigate this, poor captive housing harms males' success with females. In one study of stocking density in Mexican fruit flies (*Anastrepha ludens*) (Díaz‐Fleischer et al. 2009), and four studies of WR ‘enriched’ housing on fruit flies (*Drosophila melanogaster*) (Dukas and Mooers 2003), zebrafish (*Danio rerio*) (Lavery et al. 2025), rats *(Rattus norvegicus)* (Mitra and Sapolsky 2012) and mink (Díez‐León et al. 2013) males raised in higher stocking densities or smaller, more barren housing conditions were less successful with females. Thus, fruit flies experiencing lower stocking density (Dukas and Mooers 2003), and WR male mink (Díez‐León et al. 2013) and fruit flies (Dukas and Mooers 2003), obtained more matings than did males housed in poorer conditions. While this could reflect increased male libido, two studies further showed that housing can affect male attractiveness per se, in mate choice tests where females can choose freely. In choice arenas, female rats preferred to spend time near WR‐raised males over males raised in conventional barren cages (Mitra and Sapolsky 2012); and in T mazes, female zebrafish similarly preferred to spend time near WR males over males raised in barren tanks (Lavery et al. 2025).

This study investigated whether housing quality affects mate attractiveness in other species, to assess the generality of such effects. We predicted that females will treat males from conventional small cages as lower‐quality, preferentially approaching and sniffing/interacting with males from WR conditions. We tested this hypothesis using mice, *Mus musculus*, an excellent model species for several reasons. First, female mice do show mate choice, preferring males who are healthy (Ehman and Scott 2002) and long‐lived (Beck and Powell 2000), and—since mice sing during courtship, rather like birds only in ultrasound (e.g., Sales and Pye 1974)—who sing complex courtship songs (Hammerschmidt et al. 2009; Musolf et al. 2010). Second, mating female mice with non‐preferred partners is known to compromise their reproduction: they build poor quality nests, produce fewer litters and have lighter pups with reduced growth rates (Drickamer et al. 2000, 2003). Third, housing quality is known to affect mouse welfare: compared to WR housing, small, barren cages can increase anxiety, impair thermoregulation and compromise mouse health (Cait et al. 2022). Fourth, female mice can distinguish between differentially raised, WR or conventionally housed, female conspecifics (Adcock et al. 2021, Kitchenham et al. 2022). Fifth, females' housing conditions influence their reproductive success. For example, females housed in WR conditions have fewer abortions and stillbirths, larger litters, heavier pups and increased litter survivability compared to those conventionally housed in small lab cages (e.g., Weber and Olsson 2008; Gaskill and Pritchett‐Corning 2015; Garner et al. 2016; Leidinger et al. 2019; Meikle et al. 2020). Working with mice also allowed us to refine previous methods used to study housing quality effects on mate choice. We thus used differentially housed females to ensure that any preference for males did not simply reflect novelty effects. We induced and assessed oestrous to ensure sexual motivation, and also recorded courtship singing to check that male–female interactions were sexual in nature. We used a design that involved mate choice by females based on attractiveness (males having little opportunity to initiate physical interaction), with data being collected blind to housing quality to reduce chances of unconscious bias (cf. e.g., Day and Altman 2000). We also assessed several male attributes to examine which might reflect any effect of housing quality. Finally, after the main experiment, females were housed with WR or conventionally‐housed males and allowed to breed, opportunistically allowing us to assess reproductive success.

## 2 Methods

### 2.1 Ethical Approval

Experiments were conducted with approval from the University of Guelph Animal Care Committee (AUP #4570), and complied with Canadian Council of Animal Care guidelines (CCAC); reporting also complies with ARRIVE 2.0 guidelines (Percie du Sert et al. 2020).

### 2.2 Animals and Housing

Our broader research programme concerned how housing quality affects reproduction, and so we used mice of the DBA/2 strain: an inbred strain that in typical conventional housing conditions exhibits both stereotypic behaviour and small litter sizes (Charles River Laboratories 1999; Nip 2018; The Jackson Laboratory 2024). All were purchased from Charles River (Raleigh, North Carolina) at 3–4 weeks old. A request was made for delivery in sister pairs (two female siblings) and brother pairs (two male siblings), each from different litters to ensure all were unrelated.

On arrival as weanlings, mice were randomly assigned to WR or conventional housing (CH) treatments. For the first brother pair, the first male captured from the shipping box was placed in a WR cage, and the other in CH; for the next, the first caught male was placed in CH, and the other was assigned a WR cage; and so on: an alternating procedure that minimized biases. After a week of acclimation to their new cages, males were paired with adult ovariectomized (OVX) mice who would be their cagemates. These were surplus from another lab, and provided social contact without leading to fighting or offspring production (Gaskill and Pritchett‐Corning 2015). Female sister pairs were also randomly differentially housed (half the sister pairs WR, half CH) using a random number generator in Excel. Females were housed in pairs as they are social; and as sisters as this can enhance offspring survival through cooperative allo‐parenting (cf. König 1993; Green et al. 2023).

**Figure 1:**
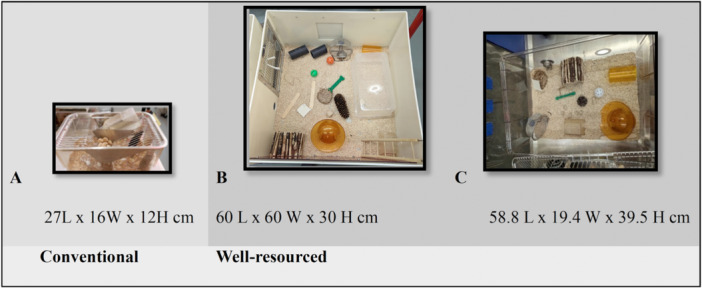
Images of cage types (approximately to scale). (A) Conventional ‘shoebox’ laboratory mouse cage, with a paper cup and two types of nest material. (B and C) Top view of the two types of well‐resourced cage, showing ‘enrichment’ items (two types of nesting material also being present in attached ‘annex’ cages not pictured here). Cage type B was used for all WR mice in the pilot study, and for WR females in the main study. Cage type C was used for WR males in the main study (see measurements for precise sizes).

Conventional housing comprised transparent polyethylene laboratory cages (27 L × 16 W × 12 H cm, Allentown Inc.), with corncob bedding (Envigo, Mississauga, Ontario, Canada), a paper cup and two types of nesting material (crinkled paper strips and cotton pads) (Figure 1A). Our WR cages (known to reduce mouse anxiety, depressive‐like behaviour and stereotypic behaviour [cf. Nip 2018; MacLellan 2023]) were either made of opaque plastic with a transparent red window (60 L × 60 W × 30 H) (Figure 1B; pilot males; females of both studies) or of transparent polyethylene (58.8 L × 19.4 W × 39.5 H cm, Allentown Inc.) (Figure 1C; main study males). All WR cages were uniformly equipped with various enrichments. These included an igloo shelter, running wheel, plastic hut and black polyvinyl chloride tunnel (all Bio‐Serv, supplied by Fisher Scientific, Ottawa, Ontario, Canada); shredded paper nesting material (Bed‐r′Nest: The Andersons) and nesting squares (Nestlets: Ancare); paper cups (including hanging cups), popsicle sticks and chewing wooden blocks (local suppliers, ON, Canada); wooden climbing structures (Amazon, Canada); a nest box (custom‐built with polyvinyl box: local supplier, ON, Canada); recycled cardboard tubes, egg cartons and cardboard boxes (all locally collected); and cloth hammocks made from repurposed socks, and blankets, along with pinecones (locally collected). All items were autoclaved before use.

A detachable conventional annex cage was also attached to each WR cage, via a tunnel: WR mice were trained to enter this to receive treats, so as to be retained there during cage‐cleaning, or easily caught there, in a low‐stress way (MacLellan 2023), when handling was needed. Routine daily health monitoring ensured that mice were free from any disease. Handling (for cage‐cleaning, health monitoring and restraint) involved a cup or tunnel and habituation to ‘3D handling’ technique (Marcotte et al. 2021), again all to minimize stress (Gouveia and Hurst 2017). The room was kept at 21°C ± 1°C and 35%–55% relative humidity, on a reverse 14L:10D hour light cycle (lights off at 07:00 and on at 17:00). Food (Harlan Teklad, Global Diet 18% protein) and water were given *ad libitum*.

#### 2.2.1 Pilot Study

The study was conducted in two phases: a pilot and a main study. The small‐scale pilot was used to check the feasibility of planned methods and identify any potential issues before proceeding with the full‐scale main study. This phase opportunistically used available animals from our research programme studying maternal care. As a result, numbers were limited, and animals differed in experience beyond housing quality: some had mating experience and had even fathered pups, while others were virgins who had never been exposed to potential mates. Adjustments were subsequently made based on this pilot, helping ensure that the main study was well‐designed and smooth to run.

Twelve DBA/2 males were used: eight unrelated 6‐month olds, and four 2‐month old F1 offspring from different litters. Twenty‐two DBA/2 females were used: eight sister pairs, 6‐months old, and six 2‐month old F1 offspring. These female F1 offspring consisted of two sister pairs from different litters and two individual females, each paired with DBA female pups from different litters (not used as test subjects), tail‐marked for identification. Of the 6‐month olds, half had already fathered or borne pups, while the rest had only mating experience but no pups. The F1 offspring had never been exposed to the opposite sex post‐weaning. For mate choice tests, unrelated, unfamiliar males and females with similar reproductive experiences were run in each trial. Each male dyad, consisting of one WR and one CH male (unrelated, but with similar past experience), was exposed to one WR and one CH female during each test (see Section 2.3).

#### 2.2.2 Main Study

For the main study, sample sizes were doubled: 12 DBA/2 sister pairs and 12 DBA/2 brother pairs. They had yet to be used in our maternal care trials; consequently, all were reproductively naive. Furthermore, mate choice was therefore followed by full mating for all the females, and for half the males.

During each mate choice test, one sister pair was exposed to one male dyad consisting of one WR and his CH brother (unrelated to the sister pair). This differed from the pilot, where each male dyad was presented to one WR sister pair and one CH sister pair; and where WR and CH males per dyad were unrelated. Furthermore, all mice were now given green vegetation (spinach, broccoli and lettuce) after a week post‐arrival until 12 weeks of age, since this boosts reproduction in caged California voles (*Microtus californicus*) (Nelson et al. 1983; Rissman and Johnston 1986). As a final difference, males were also now later phenotyped for two testosterone‐sensitive traits (see below).

### 2.3 Mate Choice Tests

**Figure 2:**
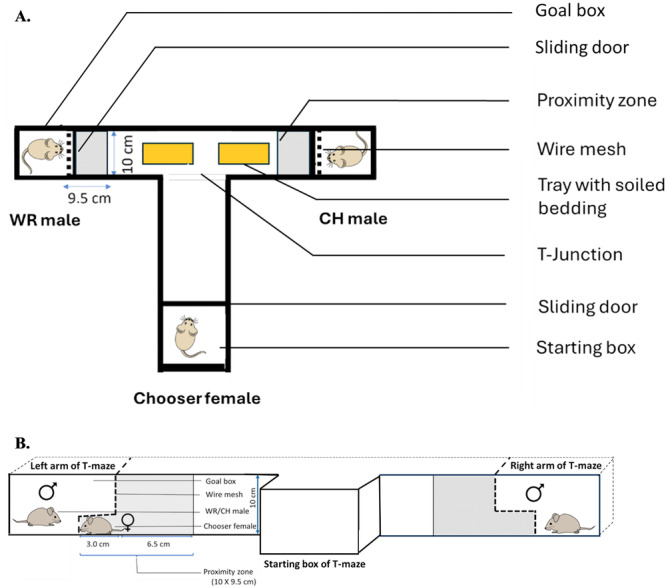
T‐maze apparatus used in mate choice test. (A) Schematic top view. (B) Schematic side view of T‐maze. Each proximity zone is shaded in grey. The mesh at the entrance of male goal box was bent inwards at the lowerhalf (extending into the goal box up to 3.0 cm) to facilitate female–male interaction (and potentially even mating).

A T‐maze was used (see Figure 2A,B), inspired by the three‐compartment apparatus used by Mitra and Sapolsky (2012) to test mate choice in rats, and resembling the T‐mazes used for evaluating partner preference in other species (e.g., ferrets: Paredes and Baum 1995; cf. Paredes 2009). We attempted a design that would permit mating, if females chose. Foam boards were also placed on all four sides to soundproof the T‐maze from external noise.

Each sister pair was first exposed to soiled bedding for 3 consecutive days, from both the cages of the males they would be exposed to in mate choice tests. This was to induce oestrous via the Whitten effect (Whitten 1956), and to pre‐familiarize females with the test males' odours. To ensure that urine of approximately equal amounts and freshness was presented from both males, given that urination may be less dense in the large WR cages, an ultraviolet pet urine detector was used to choose bedding with a high concentration of urine.

During the mate choice test, each female was placed in the starting box and after 5 min the sliding door was opened. The runway led her to a T‐junction; here, soiled bedding samples from the respective males' cages had been placed at the entrance of each arm, close to the proximity zone, to give her olfactory cues (cf. e.g., Raveh et al. 2014). At the end of each arm, each goal box held a male: one WR or one CH (the entrance of each goal box being at first closed using a sliding door). Each mate choice test began once the female had visited both males' goal boxes, while the doors of the goal boxes remained closed, to ensure she was aware of both males before recording started. The female was then brought back to the starting box, and after 5 min the sliding doors for the starting box and both the males' goal boxes were opened simultaneously. The female could then explore both arms freely. When she reached either goal box, she could now readily interact with its male, being separated from him by wire mesh (Figure 2B) that allowed exchange of visual, olfactory, auditory and tactile cues, and even mating if she chose. Each trial lasted for 30 min (modified from Drickamer et al. 2000).

All tests were observed remotely in real time (on a monitor connected to a video camera to avoid effects of human presence), as well as being video recorded. In addition, using a bat detector (Echometer Touch 2 Pro: Bat detection and recording device, Wildlife Acoustics, Inc.), the ultrasound vocalizations (USVs) produced during interaction were listened to. At the end of each test, vaginal lavage was also used to confirm that the female had been in oestrous. Although all females had been previously habituated to the vaginal lavage procedure (Marcotte et al. 2021), subjects were rewarded with treats such as oat cereal and slivered almonds after each lavage experience. To collect lavage from the vaginal canal, mice were removed from their cage, placed on the cage hopper with rear end facing the handler, and restrained by firmly grasping the tail while elevating the hindquarters (McLean et al. 2012). Approximately 0.1 mL of phosphate buffered saline was drawn into the pipette and the tip gently inserted into the vaginal orifice and the saline was flushed in and out of the vagina two to three times. A small drop of the sample was then placed evenly on a clean microscopic slide in a thin smear and allowed to air dry. Smears were fixed with methanol and then stained with modified Wright‐Giemsa stain (Sigma‐Aldrich) (e.g., Cora et al. 2015). The slides were examined immediately using a compound light microscope to determine the cell types present. Photomicrographs were taken at the time of analysis for documenting vaginal cytology. Females in oestrous having predominantly cornified epithelial cells in their smears (Byers et al. 2012). If a female was not in oestrous, her test was repeated with the same males the following day.

Before each subsequent mate choice test, the apparatus was cleaned with 70% ethanol to remove any olfactory cues. The position of the males from different housing conditions was also alternated between trials to prevent confounds between arm location and male housing quality.

All videos were then scored, blind to the housing conditions of the mice. The total time each female spent in proximity zone of either male (see Figure 2B), and the time she spent interacting with him (sniffing or making voluntary contact with her nose through the mesh) was recorded using a stopwatch.

### 2.4 Male Trait Assessment

#### 2.4.1 Body Weight

On the day of the test, before the mate choice began, the males were weighed to the nearest milligram by placement on a weighing scale.

#### 2.4.2 Tests Weight and Anogenital Distance (Main Study Males Only)

After mating and reproduction (see below), 3 months later, males were humanely euthanized. This allowed AGD and testis weight to be assessed: both testosterone‐sensitive traits. A research assistant blinded to the hypothesis assigned random codes to each, while two additional researchers, blinded to housing conditions, conducted the measurements. Testes were removed from scrotal sacs, cleaned of epididymis and any fat tissues, and weighed in pairs (to four decimal places in grammes) on a laboratory balance. Using calipers, AGD was measured as the distance from the centre of the anus to the base of the penis, and the average of the two measurements taken by the two researchers was used.

### 2.5 Reproductive Success of Differentially Housed Male‐Sister Pair Trios (Main Study Subjects Only)

Immediately after the main study's mate choice tests, each pair of sisters was housed with a similarly reared male (one of those they had been exposed to during test). Thus, WR sister pairs were now trio‐housed with their familiar WR male, while CH sister pairs were trio‐housed with their familiar CH male (regardless of who they had preferred in their test). To create these trios, mice were housed in a new cage with the male being placed first, followed by the two females after 30 min to allow the male to explore and mark his territory in the cage. They were observed in person for 2 h after placement of the females for signs of mating or aggression. Successful mating was scored when the entire mating sequence was observed—courtship, intromission and ejaculation (Oboti et al. 2014; Nicolakis et al. 2020; Preguiça 2017). Trios then remained co‐housed for 21 days.

Pregnancy was assessed by checking each female for an increase in abdominal size, beginning 10 days after the first observed mating (Pang et al. 2014), until 21 days post‐removal of the males from their cage. Latency to pregnancy (defined as the time from first being trio‐housed to first being unambiguously pregnant) was recorded in days. Pregnancy was considered successful if the females gave birth to pups. Litter size at birth, number of pups weaned, survivability rate of pups, average pup birth weight and average pup weaning weight were also recorded.

### 2.6 Statistical Analyses

#### 2.6.1 Mate Choice Data

Behavioural data were analyzed through generalized linear mixed‐effects models using R (R version 4.1.3, R Core Team, 2021). The fixed effects consisted of male housing quality, female housing quality and their interaction. Age and experience differences in the pilot study, along with familial relatedness (e.g., between father and sons), could not be incorporated due to the small sample size. Random effects consisted of male dyad (nested in female housing). Analyses focused on two primary outcome variables: the total time spent (i) in the proximity zone and (ii) sniffing/interacting with males. Response values for the sister pairs were averaged for ease of analyses (and since their values were non‐independent). Data were log‐transformed whenever needed to meet assumptions (normality and homogeneity of residuals). Any significant interaction effects were explored by splitting the data by female housing.

#### 2.6.2 Male Trait Data

Paired *t*‐tests (where each ‘pair’ was a male dyad) were run to assess any effects of housing quality on male body weight, testis weight and AGD. One male had extremely small testes and hence was excluded as an outlier when statistically analyzing testis weight.

#### 2.6.3 Reproductive Data

To evaluate the impact of housing conditions on reproductive success of the trio‐housed mice, Fisher's exact tests were performed for successful mating and pregnancies (scored as yes/no per trio). Wilcoxon rank sum tests were also used to compare, across housing types, latencies to pregnancy, number of pups born and weaned, pup survivability rate, average birth weight, o and average pup weaning weight of pup (with one value per trio).

## 3 Results

All females were in oestrous for the test, and all mate choice tests and every male–female interaction involved USVs, confirming sexual motivations.

### 3.1 Results for Pilot Study

#### 3.1.1 Total Time Spent in Proximity Zone

**Figure 3:**
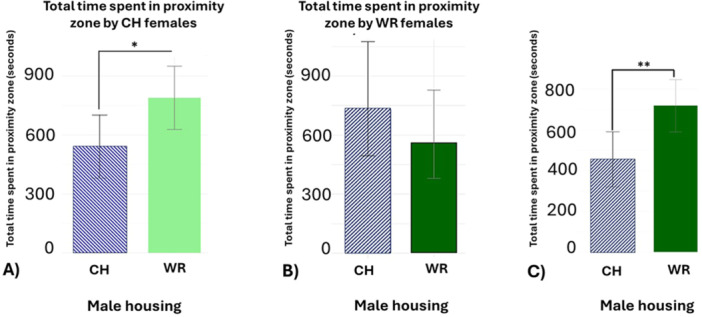
Mate preference based on the total time females spent in males' proximity. Means ± standard errors are presented. (A) Time spent in proximity zones by CH females in the pilot study. (B) Time spent in proximity zones by WR females in the pilot study (Note: for WR females, although raw data are shown in the figure, analyses were performed on log‐transformed data to meet assumptions of normality and homogeneity of residuals). (C) Time spent in proximity zones by all females in the main study. *p ≤ 0.05, **p ≤ 0.01.

Analyses revealed a significant interaction between male housing and female housing (*F*_1, 15_ = 7.11, *p* = 0.02) for time spent in the proximity zone: effects of male housing on approach by females varied with female housing type. When explored by splitting data by female housing, significant differences in time spent in WR versus CH males' proximity zones were observed in CH females (*F*_1, 10_ = 8.38, *p* = 0.02; see Figure 3A) but not WR females (*F*_1, 10_ = 1.50, *p* = 0.23; see Figure 3B).

#### 3.1.2 Total Time Spent Sniffing/Interacting With Males

**Figure 4:**
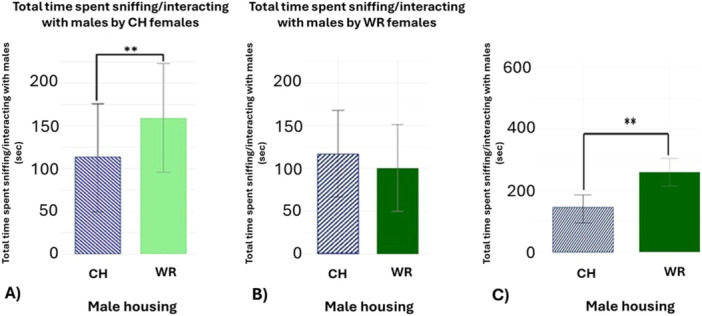
Mate preference based on the total time females spent sniffing/interacting with males. Mean ± standard errors are presented. (A) Time spent sniffing/interacting with males by CH females in the pilot study. (B) Time spent sniffing/interacting with males by WR females in the pilot study (Note: again, for WR females, although raw data are shown in the figure, statistical analyses were performed on log‐transformed data to meet assumptions). (C) Time spent sniffing/interacting with males by all females in the main study. **p ≤ 0.01.

When analyzing time spent sniffing/interacting with males, analyses again revealed a significant interaction between male and female housing (*F*_1, 10_ = 5.47, *p* = 0.04). Split by female housing, CH females spent more sniffing/interacting with WR males than CH males (*F*_1, 5_ = 33.197, *p* = 0.002; Figure 4A), whereas WR females did not (*F*_1, 5_ = 0.42, *p* = 0.54; Figure 4B).

#### 3.1.3 Housing Effects on Body Weight of Males

There was no significant difference in mean weights between WR (31.2 ± 1.29 g) and CH (34.4 ± 3.87 g) males (paired *t*_5_ = −0.99, *p* = 0.37).

### 3.2 Results for Main Study

#### 3.2.1 Total Time Spent in Proximity Zone

Analyses of total time spent in proximity zones by females revealed a significant effect of male housing quality (*F*_1, 20_ = 12.65, *p* = 0.002): females spent significantly more time with WR males than CH males. In contrast, neither the effect of female housing (*F*_1, 20_ = 0.76, *p* = 0.39) nor the interaction between male and female housing (*F*_1, 20_ = 1.295, *p* = 0.27) was significant (Figure 3C).

#### 3.2.2 Total Time Spent Sniffing/Interacting With Males

In terms of time spent sniffing and interacting, the effect of male housing was also significant (*F*_1, 20_ = 20.29, *p* = 0.002): females spent significantly more time sniffing/interacting with WR males (Figure 4C). Again, neither the effect of female housing (*F*_1, 20_ = 0.64, *p* = 0.81) nor the interaction between male and female housing (*F*_1, 20_ = 0.21, *p* = 0.65) was significant.

#### 3.2.3 Housing Quality and Male Physical Characteristics

There was no significant difference in mean body weight between WR and CH males (paired *t*_11_ = −1.1028, *p* = 0.29), nor in mean AGD values (paired *t*_11_ = −1.109, *p* = 0.298). Mean testes weight tended to be smaller in WR than CH males (paired *t*_10_ = −1.9378, *p* = 0.081); and this difference remained even after accounting for the outlier (see Table 1).

**Table 1:** Morphological parameters in males from WR and CH housing (main study).

Parameter	WR (mean ± SE)	CH (mean ± SE)	*t* (df)	*p*
Body weight (g)	27.2 ± 0.724	28.4 ± 1.07	*t*_11_ = −1.103	0.290
AGD (mm)	13.596 ± 0.841	13.95 ± 0.191	*t*_11_ = −1.109	0.298
Testes weight (g)	0.2049 ± 0.005	0.2168 ± 0.005	*t*_10_ = −1.938	0.081

### 3.3 Reproductive Success of Differentially Housed Male‐Sister Pair Trios

When each male was subsequently housed with two sisters from the same housing condition as himself, all females mated within the first 2‐h observation window. There was thus no significant difference between WR and CH trios for rates of mating. However, 6/6 WR trios gave birth to pups, while only 2/6 CH trios did: a statistical trend (Table 2). As a consequence, WR trios weaned in total over twice as many pups as CH trios (19 vs*.* 9), although this result was not significant and so should be interpreted with caution (Table 3). There was no significant difference in latencies to pregnancy, numbers of pups born, number of pups weaned, survivability of pups, average pup birth weights, nor average pup weaning weights between WR and CH trios (Table 3).

**Table 2:** Rates of successful mating and pregnancy.

Treatment	CH trios (*n* = 6)	WR trios (*n* = 6)	Fisher's exact test *p*
Number of trios where matings led to pregnancy	6	6	1
Number of trios giving birth	2	6	0.061

**Table 3:** Other reproductive parameters for WR and CH trios.

Outcome	CH trios	WR trios	Test statistic (*W*)	*p*
Latency to pregnancy (days)	17.4 ± 2.81	18.3 ± 2.87	17	0.93
Number of pups born	6.50 ± 1.50	4.33 ± 1.33	8.5	0.5
Number of pups weaned	4.50 ± 0.50	3.17 ± 1.33	8	0.6
Survivability of pups (%)	75.00 ± 25.00	52.43 ± 16.65	8.5	0.5
Average pup birth weight (g)	1.21 ± 0.11	1.36 ± 0.04	5	0.87
Average pup weaning weight (g)	8.82 ± 0.92	10.1 ± 0.49	6	1

## 4 Discussion

This study aimed to investigate the effects of housing quality on male attractiveness to females. To our knowledge, it is the first to investigate how housing quality affects female mate choice and male attractiveness in mice. The following sections discuss the degree of support for the hypothesis, this study's limitations, directions for future research on both mice and other species, and the practical and welfare implications of using WR housing to improve male attractiveness in zoos.

Our initial opportunistic pilot study provided only partial support for the hypothesis that housing quality affects male attractiveness, doing so for CH females but apparently not WR females. However, this was not a pattern predicted by theories of sexual selection, which typically propose that choosiness should *decline* with lower female quality (e.g., Dougherty 2023). We therefore suspect this apparent discrepancy was an artefact of low statistical power (just 12 males being used, 6 from each housing type) and/or the idiosyncrasies of our heterogeneous subjects who varied in age, genetic background, sexual experience and reproductive experience (and in ways that could not be statistically controlled for nor counter‐balanced with housing quality). Furthermore, the more powerful, purpose‐designed main study—featuring double the number of males, pairs of brothers matched for genetics while differing in housing quality, and no uncontrolled for variation in age or reproductive experience—*did* strongly support our hypothesis via patterns that were now similar for both CH and WR females. This revealed an approximately 50% increase in female proximity to WR males over CH, and a near doubling of their interaction with WR males over CH. The subsequent trend increase in successful pregnancies for the WR trios over CH trios was also further consistent with the hypothesis.

Such findings make theoretical sense given adaptive reasons why females should prefer low‐stress males, and evidence for such preferences in free‐living wild animals (reviewed in Section 1). They are also consistent with the five previous findings of housing quality increasing male attractiveness/breeding success, from two insect, one fish and two mammal studies. Collectively, all seven studies thus demonstrate that poor housing quality can significantly reduce male attractiveness and/or reproductive success in scenarios where females have choice. This has practical, and perhaps ethical, implications for zoos and conservation captive breeding. However, before discussing these, we consider some limitations of our study.

Our study's main limitation was that we could not ascertain which cues guided female choice. Male body weight and two testosterone‐sensitive traits (testis weight and AGD) did not vary with housing quality, and thus could not explain the effect of housing quality on male attractiveness. Potential attributes to explore in future work could therefore include glucocorticoid levels, levels of anatomical symmetry, song quality, and other cues known to influence male attractiveness in other species. Preliminary data from our lab do suggest that WR sing more readily than CH males (Cai and Mason in preparation), but whether such songs are also more attractive now requires playback studies. Stereotypic behaviours should also be assessed, as in a study of WR and CH farmed mink this was the key predictor of the reduced mating success of CH males (Díez‐León et al. 2013).

The second key limitation is that our reproductive data require caution, partly because of the small ‘N’ (just six trios per housing quality), and partly as they cannot differentiate between housing effects on male attractiveness, housing effects on male libido (e.g., as in stallions; Stout 2005), and housing effects on the females themselves. This last is important as several studies of mice (e.g., Weber and Olsson 2006; Gaskill and Pritchett‐Corning 2015; Leidinger et al. 2019) as well as other species (e.g., blue foxes, *Alopex lagopus* [Pyykönen et al. 2010], Southern white rhinoceros, *Ceratotherium simum simum* [Metrione 2010] and tigrinas, *Leopardus tigrinus* [Moreira et al. 2007]) show that females in higher‐quality housing conditions have improved reproductive success. Future replicate studies should therefore use a larger sample size, collect data on additional variables such as latency to mate, assess the potential role of mate preference in mating outcomes and also, replicate the seminal mouse mate choice study of Drickamer et al. (2000, 2003) to assess how mate preferredness affects female reproduction.

### 4.1 Practical and Welfare Implications of Using Higher‐Quality Housing to Improve Male Attractiveness

Analyses of the success of Association of Zoos and Aquariums breeding programmes have showed that only 20% of genetically recommended pairings (of 554 analyzed) resulted in successful reproduction (Faust et al. 2019). While the causes are many, including human action or inaction, they may well include ‘biological issues like… mate incompatibility’ (Faust et al. 2019). Given evidence that providing non‐preferred mates (e.g., mice: Drickamer et al. 2000; pygmy rabbits, *Brachylagus idahoensis*: Martin and Shepherdson 2012), or selecting mates solely on pedigree (e.g. stripe‐faced dunnart, *Sminthopsis macroura*: Parrott et al. 2019), may reduce conception rates, the link between housing quality and male attractiveness could help explain such figures, because they suggest that sub‐optimal male housing will increase chances that females reject them as mates.

Turning to welfare, improved male housing quality would obviously help improve male well‐being, but it could potentially have further welfare benefits for females and their offspring too. First, when reproduction is poor in endangered species, assisted reproductive techniques are sometimes proposed as solutions (e.g., Kraemer et al. 1976; Sandler et al. 2021). But improving male welfare may remove the need for such invasive (and technologically complex) interventions. Second, improved male attractiveness might improve female welfare, if being paired with unattractive males causes them stress (Mellor and Mason 1994). After all, in both Gouldian finches (*Erythrura gouldiae*) and red grouse (*Lagopus scotica*) females show elevated levels of circulating corticosterone if paired with non‐chosen mates (Griffith et al. 2011; Mougeot et al. 2016). Third, offspring could benefit via greater maternal investment when females are paired with more attractive males. For example, female mice mated with WR or more preferred males then show enhanced maternal care and better‐growing pups, potentially with better offspring survivability, compared to females mated with non‐preferred or CH males (Drickamer et al. 2000, 2003; Mashoodh et al. 2012). Likewise, female zebra finches mated with attractive males exhibit increased investment in egg mass, and have better‐growing offspring, than females mated with unattractive males (Gilbert et al. 2006). Thus, enhanced infant welfare may be another welcome side‐effect of raising their fathers in higher‐quality housing.

Overall, this study illustrates the value for conservation captive breeding of working experimentally with model species, in controlled environments, and with decent sample sizes (cf. e.g., Tung et al. 1981; Díez‐León et al. 2013; Leonelli and Ankeny 2013). It also illustrates the potential benefits for zoos of understanding natural mating systems: an evolutionary perspective that may help resolve some current conservation challenges (cf. Schulte‐Hostedde and Mastromonaco 2015). More specifically, these findings reiterate the thesis of Carlstead and Shepherdson's (1994) now classic paper that improving overall animal welfare could enhance the success of captive breeding programmes.

## 5 Conclusions

In summary, male mice from WR housing were preferred over conventionally‐housed males. Furthermore, when animals were housed together to permit mating trials, WR trios tended to have more successful pregnancies. Together, our findings suggest that enhancing housing quality could improve male attractiveness and thence reproductive success. This highlights the value of working with model species; emphasizes the need for WR housing containing species‐specific ‘enrichments’; and perhaps will further encourage continued improvements in the welfare of the captive endangered wildlife, because this could enhance not only individual animal welfare but also the broader objectives of successful conservation breeding. Further research is now essential to explore the specific traits or mechanisms through which housing quality impacts male breeding success; to investigate these effects in other taxa in more controlled conditions; and to understand their implications for maternal investment.

## Author Contributions

Prathipa Anandarajan and Georgia Mason developed the concept. Prathipa Anandarajan and Jiayi Cai carried out the tests and collected all data. Prathipa Anandarajan, Jiayi Cai and Georgia Mason carried out the statistical analysis and drafted the original text. Prathipa Anandarajan wrote the original MS, with Georgia Mason editing. All authors contributed to the review and editing of the final manuscript.

## Ethics Statement

The authors confirm that the mate choice experiment was conducted with approval from the University of Guelph, Animal Care Committee (AUP #4570) and complied with Canadian Council of Animal Care guidelines.

## Conflicts of Interest

The authors declare no conflicts of interest.

## Data Availability

Data are available from the University of Guelph Research Data Repositories (https://doi.org/10.5683/SP3/8ET1RE).

## References

[zoo70076-bib-0001] Adcock, A. , E. Choleris , M. Denommé , et al. 2021. “Where Are You From? Female Mice Raised in Enriched or Conventional Cages Differ Socially, and Can Be Discriminated by Other Mice.” Behavioural Brain Research 400: 113025. 33249072 10.1016/j.bbr.2020.113025

[zoo70076-bib-0002] Adrian, O. , G. Dekomien , J. T. Epplen , and N. Sachser . 2008. “Body Weight and Rearing Conditions of Males, Female Choice and Paternities in a Small Mammal, *Cavia aperea* .” Ethology 114, no. 9: 897–906.

[zoo70076-bib-0004] Beck, C. , and L. A. Powell . 2000. “Evolution of Female Mate Choice Based on Male Age: Are Older Males Better Mates?.” Evolutionary Ecology Research 2: 107–118.

[zoo70076-bib-0005] Byers, S. L. , M. V. Wiles , S. L. Dunn , and R. A. Taft . 2012. “Mouse Estrous Cycle Identification Tool and Images.” PLoS One 7, no. 4: e35538. 22514749 10.1371/journal.pone.0035538 PMC3325956

[zoo70076-bib-0091] Cai, J. , and G. Mason . in preparation. Courtship singing by male mice (Mus musculus) is predicted by independent effects of housing quality and testosterone‐related physical traits. Unpublished manuscript (to be submitted May 2026).

[zoo70076-bib-0006] Cait, J. , A. Cait , R. W. Scott , C. B. Winder , and G. J. Mason . 2022. “Conventional Laboratory Housing Increases Morbidity and Mortality in Research Rodents: Results of a Meta‐Analysis.” BMC Biology 20, no. 1: 15. 35022024 10.1186/s12915-021-01184-0 PMC8756709

[zoo70076-bib-0007] Carlstead, K. , and D. Shepherdson . 1994. “Effects of Environmental Enrichment on Reproduction.” Zoo Biology 13, no. 5: 447–458.

[zoo70076-bib-0008] Charles River Laboratories . 1999. “Technical Bulletin.” 2nd ed., Vol. 1 No. 1. https://www.criver.com/sites/default/files/resources/rm_rm_n_techbul_spring_99.pdf.

[zoo70076-bib-0009] Clutton‐Brock, T. H. , S. D. Albon , and F. E. Guinness . 1981. “Parental Investment in Male and Female Offspring in Polygynous Mammals.” Nature 289, no. 5797: 487–489.

[zoo70076-bib-0010] Clutton‐Brock, T. , and K. McAuliffe . 2009. “Female Mate Choice in Mammals.” Quarterly Review of Biology 84, no. 1: 3–27. 19326786 10.1086/596461

[zoo70076-bib-0011] Cora, M. C. , L. Kooistra , and G. Travlos . 2015. “Vaginal Cytology of the Laboratory Rat and Mouse: Review and Criteria for the Staging of the Estrous Cycle Using Stained Vaginal Smears.” Toxicologic Pathology 43, no. 6: 776–793. 25739587 10.1177/0192623315570339 PMC11504324

[zoo70076-bib-0012] Day, S. J. , and D. G. Altman . 2000. “Statistics Notes: Blinding in Clinical Trials and Other Studies.” BMJ 321, no. 7259: 504. 10948038 10.1136/bmj.321.7259.504 PMC1118396

[zoo70076-bib-0013] Deviche, P. J. , L. L. Hurley , H. B. Fokidis , et al. 2010. “Acute Stress Rapidly Decreases Plasma Testosterone in a Free‐Ranging Male Songbird: Potential Site of Action and Mechanism.” General and Comparative Endocrinology 169, no. 1: 82–90. 20691650 10.1016/j.ygcen.2010.07.009

[zoo70076-bib-0014] Díaz‐Fleischer, F. , J. Arredondo , and M. Aluja . 2009. “Enriching Early Adult Environment Affects the Copulation Behaviour of a Tephritid Fly.” Journal of Experimental Biology 212, no. 13: 2120–2127. 19525439 10.1242/jeb.027342

[zoo70076-bib-0015] Díez‐León, M. , J. Bowman , S. Bursian , et al. 2013. “Environmentally Enriched Male Mink Gain More Copulations Than Stereotypic, Barren‐Reared Competitors.” PLoS One 8, no. 11: e80494. 24282547 10.1371/journal.pone.0080494 PMC3839975

[zoo70076-bib-0016] Dougherty, L. R. 2023. “The Effect of Individual State on the Strength of Mate Choice in Females and Males.” Behavioral Ecology 34, no. 2: 197–209. 36998999 10.1093/beheco/arac100 PMC10047626

[zoo70076-bib-0017] Drickamer, L. C. , P. A. Gowaty , and C. M. Holmes . 2000. “Free Female Mate Choice in House Mice Affects Reproductive Success and Offspring Viability and Performance.” Animal Behaviour 59, no. 2: 371–378. 10675259 10.1006/anbe.1999.1316

[zoo70076-bib-0018] Drickamer, L. C. , P. A. Gowaty , and D. M. Wagner . 2003. “Free Mutual Mate Preferences in House Mice Affect Reproductive Success and Offspring Performance.” Animal Behaviour 65, no. 1: 105–114. 10.1006/anbe.1999.1316 10675259

[zoo70076-bib-0019] Dukas, R. , and A. Ø. Mooers . 2003. “Environmental Enrichment Improves Mating Success in Fruit Flies.” Animal Behaviour 66, no. 4: 741–749.

[zoo70076-bib-0020] Ehman, K. D. , and M. E. Scott . 2002. “Female Mice Mate Preferentially With Non‐Parasitized Males.” Parasitology 125, no. 5: 461–466. 12458830 10.1017/s003118200200224x

[zoo70076-bib-0021] Faust, L. J. , S. T. Long , K. Perišin , and J. L. Simonis . 2019. “Uncovering Challenges to Sustainability of AZA Animal Programs by Evaluating the Outcomes of Breeding and Transfer Recommendations With PMCTrack.” Zoo Biology 38, no. 1: 24–35. 30614074 10.1002/zoo.21470

[zoo70076-bib-0022] Forstmeier, W. 2002. “Factors Contributing to Male Mating Success in the Polygynous Dusky Warbler (*Phylloscopus fuscatus*).” Behaviour 139, no. 10: 1361–1381.

[zoo70076-bib-0023] Gangestad, S. W. , L. A. Merriman , and M. E. Thompson . 2010. “Men's Oxidative Stress, Fluctuating Asymmetry and Physical Attractiveness.” Animal Behaviour 80, no. 6: 1005–1013.

[zoo70076-bib-0092] Garner, J. P. , B. N. Gaskill , and K. R. Pritchett‐Corning . 2016. “Two of a Kind or a Full House? Reproductive Suppression and Alloparenting in Laboratory Mice.” PLoS One 11, no. 5: e0154966. 27148872 10.1371/journal.pone.0154966 PMC4858245

[zoo70076-bib-0024] Gaskill, B. N. , and K. R. Pritchett‐Corning . 2015. “The Effect of Cage Space on Behavior and Reproduction in Crl: CD1 (Icr) and C57BL/6NCrl Laboratory Mice.” PLoS One 10, no. 5: e0127875. 26020792 10.1371/journal.pone.0127875 PMC4447268

[zoo70076-bib-0025] Gilbert, L. , K. A. Williamson , N. Hazon , and J. A. Graves . 2006. “Maternal Effects Due to Male Attractiveness Affect Offspring Development in the Zebra Finch.” Proceedings of the Royal Society B: Biological Sciences 273, no. 1595: 1765–1771. 10.1098/rspb.2006.3520 PMC1634786 16790409

[zoo70076-bib-0093] Green, J. P. , C. Franco , A. J. Davidson , et al. 2023. “Cryptic kin Discrimination During Communal Lactation in Mice Favours Cooperation Between Relatives." Communications.” Biology 6, no. 1: 734. 10.1038/s42003-023-05115-3 PMC10349843 37454193

[zoo70076-bib-0026] Gouveia, K. , and J. L. Hurst . 2017. “Optimising Reliability of Mouse Performance in Behavioural Testing: The Major Role of Non‐Aversive Handling.” Scientific Reports 7, no. 1: 44999. 28322308 10.1038/srep44999 PMC5359560

[zoo70076-bib-0027] Griffith, S. C. , S. R. Pryke , and W. A. Buttemer . 2011. “Constrained Mate Choice in Social Monogamy and the Stress of Having an Unattractive Partner.” Proceedings of the Royal Society B: Biological Sciences 278, no. 1719: 2798–2805. 10.1098/rspb.2010.2672 PMC3145185 21288942

[zoo70076-bib-0028] Hammerschmidt, K. , K. Radyushkin , H. Ehrenreich , and J. Fischer . 2009. “Female Mice Respond to Male Ultrasonic ‘Songs’ With Approach Behaviour.” Biology Letters 5, no. 5: 589–592. 19515648 10.1098/rsbl.2009.0317 PMC2781958

[zoo70076-bib-0030] Jean‐Faucher, C. , M. Berger , M. De Turckheim , G. Veyssiere , and C. Jean . 1981. “Effects of Dense Housing on the Growth of Reproductive Organs, Plasma Testosterone Levels and Fertility of Male Mice.” Journal of Endocrinology 90, no. 3: 397–402. 7276797 10.1677/joe.0.0900397

[zoo70076-bib-0031] Jennions, M. D. , and M. Petrie . 1997. “Variation in Mate Choice and Mating Preferences: A Review of Causes and Consequences.” Biological Reviews 72, no. 2: 283–327. 9155244 10.1017/s0006323196005014

[zoo70076-bib-0032] Jones, S. D. , J. F. Wallman , and P. G. Byrne . 2015. “Do Male Secondary Sexual Characters Correlate With Testis Size and Sperm Length in the Small Hairy Maggot Blowfly?.” Zoology 118, no. 6: 439–445. 26297128 10.1016/j.zool.2015.07.003

[zoo70076-bib-0033] Kenrick, D. T. , E. K. Sadalla , G. Groth , and M. R. Trost . 1990. “Evolution, Traits, and the Stages of Human Courtship: Qualifying the Parental Investment Model.” Journal of Personality 58, no. 1: 97–116. 23750377 10.1111/j.1467-6494.1990.tb00909.x

[zoo70076-bib-0034] Khan, A. S. , J. L. Brown , V. Kumar , G. Umapathy , and N. Baskaran . 2023. “Measures of Adrenal and Gonadal Hormones in Relation to Biological and Management Factors Among Captive Red Pandas in Indian Zoos.” Animals 13, no. 8: 1298. 37106861 10.3390/ani13081298 PMC10135066

[zoo70076-bib-0035] Kirkpatrick, M. , and M. J. Ryan . 1991. “The Evolution of Mating Preferences and the Paradox of the Lek.” Nature 350, no. 6313: 33–38.

[zoo70076-bib-0094] Kitchenham, L. , B. Nazal , A. Adcock , E. Nip , A. MacLellan , and G. Mason . 2022. “Why Does Lifelong Conventional Housing Reduce the Sociability of Female Mice?” Applied Animal Behaviour Science 246: 105532.

[zoo70076-bib-0036] Koehler, N. , G. Rhodes , and L. W. Simmons . 2002. “Are Human Female Preferences for Symmetrical Male Faces Enhanced When Conception Is Likely?.” Animal Behaviour 64, no. 2: 233–238.

[zoo70076-bib-0095] König, B. 1993. “Maternal Investment of Communally Nursing Female House Mice (Mus musculus domesticus).” Behavioural Processes 30, no. 1: 61–73. 24896472 10.1016/0376-6357(93)90012-G

[zoo70076-bib-0037] Kraemer, D. C. , G. T. Moore , and M. A. Kramen . 1976. “Baboon Infant Produced by Embryo Transfer.” Science 192, no. 4245: 1246–1247. 818710 10.1126/science.818710

[zoo70076-bib-0038] Lavery, J. M. , K. Snaith , J. Pallarca , K. Raine , and G. J. Mason . 2025. “Good Welfare Is Attractive: Female Zebrafish (*Danio rerio*) Prefer Males From Complex, Well‐Resourced Conditions Over Males From Conventional Barren Laboratory Tanks.” Applied Animal Behaviour Science 286: 106603.

[zoo70076-bib-0039] Leidinger, C. S. , C. Thöne‐Reineke , N. Baumgart , and J. Baumgart . 2019. “Environmental Enrichment Prevents Pup Mortality in Laboratory Mice.” Laboratory Animals 53, no. 1: 53–62. 29788793 10.1177/0023677218777536

[zoo70076-bib-0040] Leonelli, S. , and R. A. Ankeny . 2013. “What Makes a Model Organism?.” Endeavour 37, no. 4: 209–212. 23849606 10.1016/j.endeavour.2013.06.001

[zoo70076-bib-0041] MacLellan, A. 2023. “Do Conventional Cages Make Laboratory Mice Depressed? A Framework for Identifying Animal Depression.” Doctoral diss., University of Guelph.

[zoo70076-bib-0042] Marcotte, M. , A. Bernardo , N. Linga , et al. 2021. “Handling Techniques to Reduce Stress in Mice.” Journal of Visualized Experiments 25, no. 175: e62593. 10.3791/62593 34633376

[zoo70076-bib-0043] Mashoodh, R. , B. Franks , J. P. Curley , and F. A. Champagne . 2012. “Paternal Social Enrichment Effects on Maternal Behavior and Offspring Growth.” supplement, Proceedings of the National Academy of Sciences 109, no. suppl_2: 17232–17238. 10.1073/pnas.1121083109 PMC3477388 23045657

[zoo70076-bib-0096] Martin, M. S. , and D. J. Shepherdson . 2012. “Role of Familiarity and Preference in Reproductive Success in ex Situ Breeding Programs.” Conservation Biology 26, no. 4: 649–656. 22809353 10.1111/j.1523-1739.2012.01880.x

[zoo70076-bib-0044] McLean, A. C. , N. Valenzuela , S. Fai , et al. 2012. “Performing Vaginal Lavage, Crystal Violet Staining, and Vaginal Cytological Evaluation for Mouse Estrous Cycle Staging Identification.” Journal of Visualized Experiments 67: 4389. 10.3791/4389 PMC3490233 23007862

[zoo70076-bib-0097] Meikle, M. N. , A. P. Arévalo , G. Schlapp , G. Fernández‐Graña , A. Menchaca , and M. Crispo . 2020. “Long‐Term Effect of Environmental Enrichment on Reproductive Performance of Swiss Webster Mice and Their Female Offspring.” Animals 10, no. 8: 1438. 32824668 10.3390/ani10081438 PMC7460265

[zoo70076-bib-0045] Mellor, E. L. , and G. J. Mason . 2023. “Feeding, Mating and Animal Wellbeing: New Insights From Phylogenetic Comparative Methods.” Proceedings of the Royal Society B 290, no. 1994: 20222571. 36855870 10.1098/rspb.2022.2571 PMC9975651

[zoo70076-bib-0046] Metrione, L. C. 2010. “Relationships of Social Behavior and the Captive Environment to Reproduction in Female Southern White Rhinoceros (*Ceratotherium simum simum*).” Doctoral diss., Ohio State University.

[zoo70076-bib-0047] Mitra, R. , and R. M. Sapolsky . 2012. “Short‐Term Enrichment Makes Male Rats More Attractive, More Defensive and Alters Hypothalamic Neurons.” PLoS One 7, no. 5: e36092. 22567125 10.1371/journal.pone.0036092 PMC3342313

[zoo70076-bib-0048] Moore, F. R. , E. A. S. Al Dujaili , R. E. Cornwell , et al. 2011. “Cues to Sex‐ and Stress‐Hormones in the Human Male Face: Functions of Glucocorticoids in the Immunocompetence Handicap Hypothesis.” Hormones and Behavior 60, no. 3: 269–274. 21672543 10.1016/j.yhbeh.2011.05.010

[zoo70076-bib-0049] Moreira, N. , J. L. Brown , W. Moraes , W. F. Swanson , and E. L. A. Monteiro‐Filho . 2007. “Effect of Housing and Environmental Enrichment on Adrenocortical Activity, Behavior and Reproductive Cyclicity in the Female Tigrina (*Leopardus tigrinus*) and Margay (*Leopardus wiedii*).” Zoo Biology 26, no. 6: 441–460. 19360593 10.1002/zoo.20139

[zoo70076-bib-0050] Mougeot, F. , Á. Z. Lendvai , J. Martínez‐Padilla , et al. 2016. “Parasites, Mate Attractiveness and Female Feather Corticosterone Levels in a Socially Monogamous Bird.” Behavioral Ecology and Sociobiology 70, no. 2: 277–283.

[zoo70076-bib-0051] Musolf, K. , F. Hoffmann , and D. J. Penn . 2010. “Ultrasonic Courtship Vocalizations in Wild House Mice, *Mus musculus musculus* .” Animal Behaviour 79, no. 3: 757–764.

[zoo70076-bib-0052] Nelson, R. J. , J. Dark , and I. Zucker . 1983. “Influence of Photoperiod, Nutrition and Water Availability on Reproduction of Male California Voles (*Microtus californicus*).” Reproduction 69, no. 2: 473–477. 10.1530/jrf.0.0690473 6355461

[zoo70076-bib-0053] Nevison, C. M. , J. L. Hurst , and C. J. Barnard . 1999. “Why Do Male ICR (CD‐1) Mice Perform Bar‐Related (Stereotypic) Behaviour?.” Behavioural Processes 47, no. 2: 95–111. 24896933 10.1016/s0376-6357(99)00053-4

[zoo70076-bib-0054] Nicolakis, D. , M. A. Marconi , S. M. Zala , and D. J. Penn . 2020. “Ultrasonic Vocalizations in House Mice Depend Upon Genetic Relatedness of Mating Partners and Correlate With Subsequent Reproductive Success.” Frontiers in Zoology 17, no. 1: 10. 32265997 10.1186/s12983-020-00353-1 PMC7118824

[zoo70076-bib-0055] Nip, E. 2018. “The Long‐Term Effects of Environmental Enrichment on Agonism in Female C57BL/6, DBA/2, and BALB/c Mice.” Doctoral diss., University of Guelph.

[zoo70076-bib-0056] Oboti, L. , A. Pérez‐Gómez , M. Keller , et al. 2014. “A Wide Range of Pheromone‐Stimulated Sexual and Reproductive Behaviors in Female Mice Depend on G Protein Gαo.” BMC Biology 12, no. 1: 31. 24886577 10.1186/1741-7007-12-31 PMC4038847

[zoo70076-bib-0057] Ophir, A. G. , and J. Delbarco‐Trillo . 2007. “Anogenital Distance Predicts Female Choice and Male Potency in Prairie Voles.” Physiology & Behavior 92, no. 3: 533–540. 17537467 10.1016/j.physbeh.2007.04.030

[zoo70076-bib-0058] Özener, B. 2010. “Fluctuating and Directional Asymmetry in Young Human Males: Effect of Heavy Working Condition and Socioeconomic Status.” American Journal of Physical Anthropology 143, no. 1: 112–120. 20734438 10.1002/ajpa.21300

[zoo70076-bib-0059] Pang, S. C. , J. Janzen‐Pang , M. Y. Tse , B. A. Croy , and P. D. A. Lima . 2014. “The Cycling and Pregnant Mouse: Gross Anatomy.” In The Guide to Investigation of Mouse Pregnancy, edited by B. A. Croy , A. T. Yamada , F. J. DeMayo , and S. L. Adamson , 3–19. Academic Press.

[zoo70076-bib-0060] Paredes, R. G. 2009. “Evaluating the Neurobiology of Sexual Reward.” ILAR Journal 50, no. 1: 15–27. 19106449 10.1093/ilar.50.1.15

[zoo70076-bib-0061] Paredes, R. G. , and M. Baum . 1995. “Altered Sexual Partner Preference in Male Ferrets Given Excitotoxic Lesions of the Preoptic Area/Anterior Hypothalamus.” Journal of Neuroscience 15, no. 10: 6619–6630. 7472423 10.1523/JNEUROSCI.15-10-06619.1995 PMC6578011

[zoo70076-bib-0062] Parrott, M. L. , A. Nation , and L. Selwood . 2019. “Female Mate Choice Significantly Increases Captive Breeding Success, and Scents Can be Frozen to Determine Choice, in the Stripe‐Faced Dunnart.” Applied Animal Behaviour Science 214: 95–101.

[zoo70076-bib-0063] Parsons, P. A. 1998. “Behavioral Variability and Limits to Evolutionary Adaptation Under Stress.” In Advances in the Study of Behaviour, Vol. 27, 155–180. Academic Press.

[zoo70076-bib-0064] Percie du Sert, N. , V. Hurst , A. Ahluwalia , et al. 2020. “The ARRIVE Guidelines 2.0: Updated Guidelines for Reporting Animal Research.” Journal of Cerebral Blood Flow and Metabolism 40, no. 9: 1769–1777. 32663096 10.1177/0271678X20943823 PMC7430098

[zoo70076-bib-0065] Preguiça, I. M. 2017. “The Role of the PgR+ Cells of the Ventromedial Hypothalamus (VMHvl) in Female Mice Sexual Receptivity.” Master's thesis, Universidade de Coimbra.

[zoo70076-bib-0066] Pyykönen, T. , L. Ahola , S. Hänninen , and J. Mononen . 2010. “Nest Provision Influences Reproductive Success in Breeding Blue Fox Vixens: A Preliminary Study.” Animal Welfare 19, no. 1: 101–105.

[zoo70076-bib-0067] Raveh, S. , S. Sutalo , K. E. Thonhauser , et al. 2014. “Female Partner Preferences Enhance Offspring Ability to Survive an Infection.” BMC Evolutionary Biology 14, no. 1: 14. 24450606 10.1186/1471-2148-14-14 PMC3905909

[zoo70076-bib-0068] Reynolds, J. D. , and M. R. Gross . 1990. “Costs and Benefits of Female Mate Choice: Is There a Lek Paradox?.” American Naturalist 136, no. 2: 230–243.

[zoo70076-bib-0069] Rissman, E. F. , and R. E. Johnston . 1986. “Nutritional and Social Cues Influence the Onset of Puberty in California Voles.” Physiology & Behavior 36, no. 2: 343–347. 3515374 10.1016/0031-9384(86)90027-2

[zoo70076-bib-0070] Roberts, M. L. , K. L. Buchanan , A. T. D. Bennett , and M. R. Evans . 2007. “Mate Choice in Zebra Finches: Does Corticosterone Play a Role?.” Animal Behaviour 74, no. 4: 921–929.

[zoo70076-bib-0071] Sales, G. D. , and D. Pye . 1974. *Ultrasonic Communication by Animals*. Springer.

[zoo70076-bib-0072] Sandler, R. L. , L. Moses , and S. M. Wisely . 2021. “An Ethical Analysis of Cloning for Genetic Rescue: Case Study of the Black‐Footed Ferret.” Biological Conservation 257: 109118.

[zoo70076-bib-0098] Schulte‐Hostedde, A. I. , and G. F. Mastromonaco . 2015. “Integrating Evolution in the Management of Captive Zoo Populations.” Evolutionary Applications 8, no. 5: 413–422. 26029256 10.1111/eva.12258 PMC4430766

[zoo70076-bib-0074] Sørensen, D. B. , I. M. Jegstrup , M. Ritskes‐Hoitinga , and A. K. Hansen . 2005. “Fluctuating Asymmetry in Relation to Stress and Social Status in Inbred Male Lewis Rats.” Scandinavian Journal of Laboratory Animal Science 32, no. 2: 117–123.

[zoo70076-bib-0075] Stout, T. A. E. 2005. “Modulating Reproductive Activity in Stallions: A Review.” Animal Reproduction Science 89, no. 1–4: 93–103. 16061332 10.1016/j.anireprosci.2005.06.015

[zoo70076-bib-0076] Swaddle, J. P. , and I. C. Cuthill . 1994. “Preference for Symmetric Males by Female Zebra Finches.” Nature 367, no. 6459: 165–166.

[zoo70076-bib-0077] Taylor, P. D. , and G. C. Williams . 1982. “The Lek Paradox Is Not Resolved.” Theoretical Population Biology 22, no. 3: 392–409.

[zoo70076-bib-0078] The Jackson Laboratory . 2024. “Strain: DBA/2J (000671).” https://www.jax.org/strain/000671.

[zoo70076-bib-0079] Trivers, R. L. 1972. “Parental Investment and Sexual Selection.” In Sexual Selection and the Descent of Man, edited by B. Campbell , 136–179. Aldine‐Atherton.

[zoo70076-bib-0080] Tung, K. S. , L. Ellis , C. Teuscher , et al. 1981. “The Black Mink (*Mustela vison*). A Natural Model of Immunologic Male Infertility.” Journal of Experimental Medicine 154, no. 4: 1016–1032. 6116740 10.1084/jem.154.4.1016 PMC2186489

[zoo70076-bib-0081] Weber, E. M. , and I. A. S. Olsson . 2006. “Maternal Behaviour in Laboratory Mice: The Effect of Housing Environment.” In Proceedings of the 40th International Congress of the ISAE, 272. International Society of Applied Ethology.

[zoo70076-bib-0099] Weber, E. M. , and I. A. S. Olsson . 2008. “Maternal Behaviour in Mus musculus sp: An Ethological Review.” Applied Animal Behaviour Science 114, no. 1‐2: 1–22.

[zoo70076-bib-0082] Whitten, W. K. 1956. “Modification of the Oestrous Cycle of the Mouse by External Stimuli Associated With the Male.” Journal of Endocrinology 13, no. 4: 399–404. 13345955 10.1677/joe.0.0130399

[zoo70076-bib-0083] Wielebnowski, N. C. , N. Fletchall , K. Carlstead , J. M. Busso , and J. L. Brown . 2002. “Noninvasive Assessment of Adrenal Activity Associated With Husbandry and Behavioral Factors in the North American Clouded Leopard Population.” Zoo Biology 21, no. 1: 77–98.

[zoo70076-bib-0084] Williams, G. C. 1975. Sex and Evolution. Princeton University Press.

